# The landscape of biobanks in Poland—characteristics of Polish biobanking units at the beginning of BBMRI.pl organization

**DOI:** 10.1186/s12967-021-02926-y

**Published:** 2021-06-22

**Authors:** Anna Chróścicka, Angelika Paluch, Łukasz Kozera, Małgorzata Lewandowska-Szumieł

**Affiliations:** 1 Department of Histology and Embryology, Center for Biostructure Research, Medical University of Warsaw, 61 Żwirki i Wigury St., 02-091 Warsaw, Poland; 2 Laboratory for Cell Research and Application, Center for Preclinical Research and Technology, Medical University of Warsaw, 61 Żwirki i Wigury St., 02-091 Warsaw, Poland; 3 BBMRI.pl Consortium, Warsaw, Poland; 4 BBMRI-ERIC, Neue Stiftingtalstrasse 2/B/6, 8010 Graz, Austria

**Keywords:** Biobank, Biorepository, Human samples, Biospecimen, Research, Poland, BBMRI.pl, Polish Biobanking Network

## Abstract

**Background:**

Biobanking is an area of scientific activity that is growing in strength and importance. The variety of collections combining biological samples and medical scientific information makes biobanking an indispensable tool in the development of modern medicine. In 2016, Poland, a country with one of the largest populations in Europe, joined the Biobanking and BioMolecular resources Research Infrastructure-European Research Infrastructure Consortium (BBMRI-ERIC) to facilitate access to quality-defined human disease-relevant biological resources. This push led to the development of the Polish Biobanking Network. The purpose of this paper is to present the current state of biobanks in Poland in the context of their location, nature and resources.

**Methods:**

To obtain information about and overall characteristics of Polish entities dealing with biobanking biological material, the dedicated *Information Survey* was designed. The survey was prepared in an electronic form and consisted of 53 questions—both open and closed, single and multiple choice—with some questions depending on each other. Sixty-five Polish biobanks/biorepositories participated in the survey.

**Results:**

Polish biobanks are mostly affiliated with research entities (universities—42% and research institutes—30%). The data collected indicate that a considerable number of Polish biobanks are specialized (33 units), in contrast to population-based biobanks (8 units). These biobanks are mostly focused on collecting samples from oncological (23 biobanks) and rare diseases (12 biobanks). In general, great diversity was found in the material collected. Scientists working in Polish biobanks are very open to scientific cooperation (declared by 60% of units) and sharing their collections with the international scientific environment. In terms of quality issues, most biobanks declared that their quality management system was in the process of implementation (45%) or had already been implemented (23%).

**Conclusions:**

Although biobanking in Poland is still in its infancy, the results of this study seem promising and may be valuable to the wider biobanking research community. The distribution of biobanks throughout the Polish territory, their connection with scientific and clinical units, and their involvement in research on rare diseases may contribute to an increase in the number of multicenter studies.

## Background

Recent years have shown a growing interest in the topic of broadly defined biobanking, which has significantly influenced the development of biobanking infrastructures and biomedical technologies [1–4]. There are many definitions of a “biobank”, but typically, this is an institution focused on the collection of biological material and/or associated data for many different purposes (i.e., research, diagnostic) with properly organized management and data storage IT systems [5, 6]. According to ISO 20387:2018 *Biotechnology—Biobanking—General requirements for biobanking*, a biobank is defined as a legal entity or a specific part of a legal entity that is legally responsible for all its activities and performs acquisition and storage activities along with some or all of the activities related to the collection, preparation, maintenance, testing, analysis and distribution of certain biological materials and related data for scientific research and development of medical technologies that use accumulated resources in multiple and long-term ways [7]. The definition taken from the *Quality Standards for Polish Biobanks,* created by the BBMRI.pl Consortium, indicates that a biobank is an organizational unit involved in gathering (collection, processing, storage), distributing and sharing biological material, and the data retrieved from this material are used for the purposes of scientific research [8]. Such a unit uses the collected resources in multiple and long-term ways, with specific procedures to preserve the high quality of collected resources and protect the rights of donors, as well as having an appropriate supervisory body in the form of a scientific and ethical committee for biobanking [8–10]. Biobank should meet several requirements criteria that define, e.g., the mission and the scope of its activities [8, 11, 12]. It should also be emphasized that the proper functioning of a biobank requires employing well-qualified and well-selected personnel [13]. Therefore, biobanks are institutions that provide high-quality storage conditions for biological material that can be used in a wide range of scientific studies [14]. Using biological samples obtained from biobanks can increase the credibility of research and therefore contribute to understanding the mechanisms underlying a given disease or molecular modification [15]. Thanks to a better understanding of specific disease mechanisms, clinicians in cooperation with scientists can cure patients with targeted therapies. Storage of biological material derived from diseased patients enables comparative analysis before and after treatment, often on a large scale [16].

The main goal of biobanking is to support and conduct reliable scientific research and development [14, 16]. Different types of biobanks have been distinguished depending on the type of material that is collected [14, 16, 17]. Currently, we recognize population, specialized, clinical, and mixed biobanks. The first focuses on collecting biological samples mainly from the control population (i.e., healthy volunteers) without specific inclusion or exclusion criteria. Their main goal was to verify the influence of specific factors on the chosen population and to monitor public health. The second type, specialized biobanks, can also be named disease-oriented biobanks and are focused on collecting biological material from diseased individuals; usually, sample collection takes place in the hospital. There are many types of specialized biobanks. Most studies concentrate on oncological/cardiovascular diseases, rare diseases and genetic disorders. Disease-oriented biobanks are in possession of an extremely unique material. Collecting material connected with rare “orphan” or “neglected” diseases allows scientific research requiring the use of a certain amount of material that, without biobanks, may be difficult to obtain [18]. These biobanks may have a very large influence on the development of biomedical research in different disciplines, such as personalized medicine and omics sciences [18–22]. Clinical biobanks are usually located in healthcare units. The process of conducting clinical research is inextricably linked to the production of waste material that can be collected in biobanks for scientific purposes. As a result, scientists can conduct further research on unique materials. These biobanks have become a link between the clinical units and research departments of pharmaceutical and/or diagnostic companies [23, 24]. Mixed biobanks are units that link the features of different types of biobanks, such as populations and specialized biobanks, and are focused on a specific disease that occurs in a certain population.

Because biobanks provide the opportunity to collect specific types of biological material, scientists from around the world can carry out their research much more efficiently [25]. This is due to the ability to share material between individual units. A key factor in this regard is the proper quality of the collected samples. Each biobank usually has its own procedures that are calibrated and validated for its own needs, leading to a decentralized evolution of biobanks. Until now, there have been no uniform regulations unifying activities related to proceeding broadly understood biobanking. Polish law does not provide legislative rules for the correct operation of biobanks. To ensure the best standards for obtaining, storing, and working with biological material, it is crucial to ensure the harmonization of biobanks [1, 12, 19, 26]. This phenomenon indicates the need to create networks that could standardize the quality management system for biobanks [1, 19]. One such network is the pan-European Biobanking and BioMolecular resources Research Infrastructure-European Research Infrastructure Consortium (BBMRI-ERIC), which links biobanks and information about them and their collections from all of Europe [27, 28]. The idea of BBMRI-ERIC is to create a well-organized network consisting of European biobanks that will have a properly designed and harmonized quality management system (QMS), informatics system (IT) and ethical, legal, and social implications system (ELSI) [29]. Poland, as a BBMRI.pl, has been a full member of the BBMRI-ERIC family since 2016. The main task of BBMRI.pl is to create a Polish Biobanking Network (PBN). One of the goals of the BBMRI.pl is mapping biobanks and biorepositories located in Poland and harmonizing them in accordance with the applicable pan-European regulations adapted to Polish conditions. One of the main tasks for BBMRI.pl is obtaining knowledge about biobanks in Poland and the topics associated with them. To date, the situation of Polish biobanks is not well known. To our knowledge, no prior studies have examined the development of biobanks in Poland, and no study has focused on the characteristics of Polish biobanks. As part of the BBMRI.pl Project, we have taken steps to identify units that collect biological material. These activities were the first step towards the creation of the PBN. To do this, we have developed and widely promoted the survey (named *Information Survey*) in the biomedical environment all over the country.

The aim of this paper is to show the results of that survey and present the situation of biobanks in Poland, the types of units that exist there, the types of biological material they collect, and their general characteristics. Taking into account the administrative area, Poland ranks 69th in the world and 9th in Europe [30] (in terms of population, Poland ranks 38th in the world [31] and 9th in Europe [32]), we believe that the presented data are important to the wider biobanking research community.

## Methods

To obtain information about Polish entities dealing with biobanking of biological material, the dedicated *Information Survey* was designed [33]. For the purposes of our study, a biobank was defined as ‘an entity that acquires, collects or stores biological material and associated data for future research use’. The survey was prepared in electronic form with online access only. It contained questions to obtain basic information about entities dealing with the biobanking of biological material, which, however, would allow their initial characterization. It was important that the survey contained a limited number of questions and did not take too long to complete. This design of the survey increases the chances of it being fully completed by more people involved in biobanking. The *Information Survey* consisted of 53 questions—both open and closed, single and multiple choice—some of the questions depended on each other. The survey was created to obtain information about the geographical location of the biobank. Its functioning mode included the type of collected material, the number of samples, location in the parent institution structure, the type of biobank, willingness to share deposited material, quality management and IT systems, and readiness to participate in workshops and trainings [21, 34]. Before publishing the survey, the content of questions and their scope and form were reviewed in consultation with many Polish scientists of various specialties to ensure that they were understandable. To reach the largest possible number of recipients, extensive promotional and information campaigns were carried out. Information about the project and survey was published on many websites and dedicated portals that are popular among Polish scientists. Additionally, information was sent to most Polish medical universities, faculties of biological sciences and other relevant institutes. The identification of Polish biobanks also included searching available sources, including web browsers, publication databases, and direct meetings with scientists [35]. After identifying units collecting biological material, the process of recognizing the nature of their activities was followed by direct contact with individual units and an invitation to complete the survey.

In total, approximately 400 individuals (representatives of many different units) selected as a result of the activities described above were informed by email or by phone about the survey preparation. Subsequently, invitations to participate in the survey were sent to 349 identified stakeholders. Some of them (51 units) were not interested in the project, and some of them changed or deleted their activity profile. Out of 349 sent invitations, 89 users registered in the survey system, and 65 completed the informational survey. Twenty-four participants did not complete the survey fully; hence, their responses were not included in the data analysis.

After developing the survey, data acquisition and analysis were carried out in 2017–2019, and this was part of the large project implemented in Poland and funded by grants from the Ministry of Science and Higher Education.

## Results

**Fig. 1:**
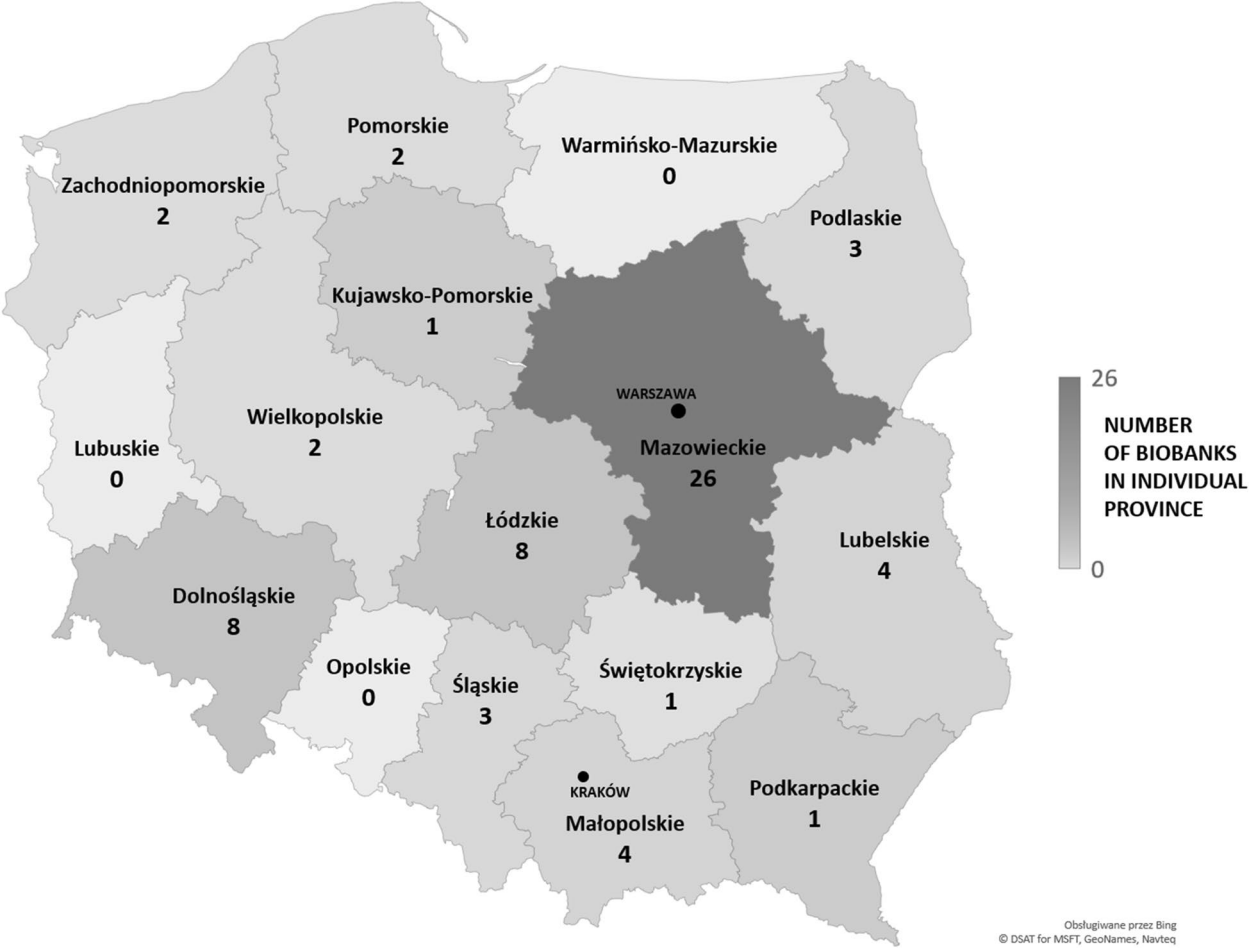
The number of biobanks in individual Polish provinces. The highest number of biobanks is located in Mazowieckie Province. While the lowest number is in Kujawsko-Pomorskie, Świętokrzyskie, and Podkarpackie Provinces. There is no identified biobank in Warmińsko-Mazurskie, Lubuskie and Opolskie Provinces

The majority of the analyzed units were in Mazowieckie (26 biobanks), Dolnośląskie (8 biobanks) and Łódzkie (8 biobanks) provinces. Fewer biobanks were located in Lubelskie (4 biobanks), Małopolskie (4 biobanks), Podlaskie (3 biobanks), Śląskie (3 biobanks), Wielkopolskie (2 biobanks), Pomorskie (2 biobanks), Zachodniopomorskie (2 biobanks), Kujawsko-Pomorskie (1 biobank), Podkarpackie (1 biobank) and Świętokrzyskie (1 biobank) provinces. There were three provinces in which biobanks have not been identified. The geographical distribution of all identified units (biobanks and biorepositories) is shown in Fig. 1.

Regarding the origin of the biological material, 60 out of 65 institutions that took part in the survey (92%) declared that they have collected human biological material—both samples and/or data related to them. Additionally, 9 out of 65 identified units (14%) reported storage of animal biospecimens, 9% reported storage of biological material of microbe origin, and 5% reported storage of plant biosamples. In this study, we present only the results from 60 biobanks/biorepositories that collect human biological material and data connected with it.

**Fig. 2:**
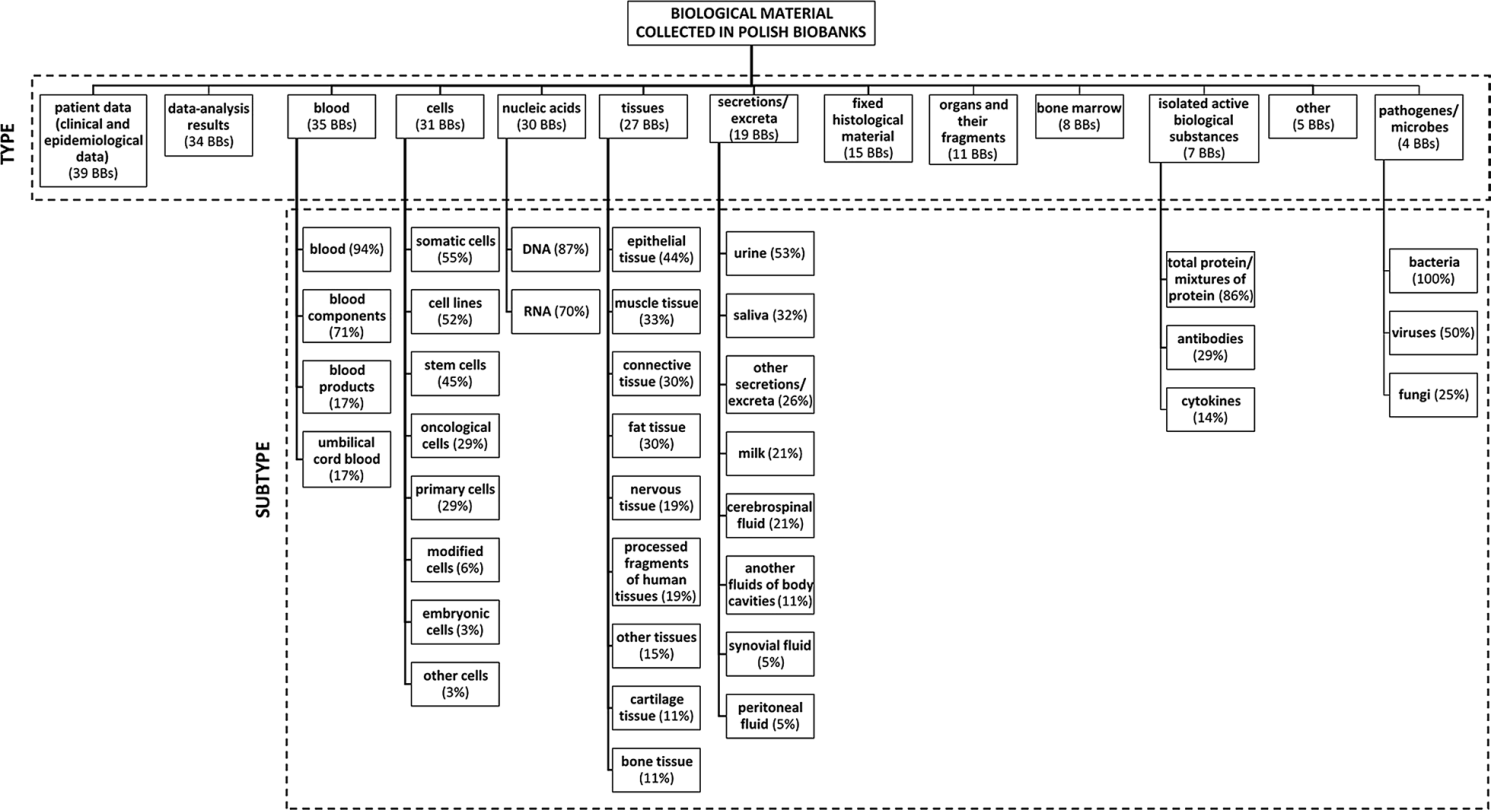
Different types of biological material (BM) collected in Polish biobanks (BBs). The number of BBs collecting a specific type of BM is shown in brackets. Each of the BM subtypes was calculated in reference to the number of BBs that are specialized in collecting the specific type of BM. A single biobank can collect one or some types of BM

Considering human biological material collections, we asked about the type of biological specimen stored in biobanks. The respondents were asked what type of human biological material their biobanks focused on. They could choose among patient input data, the results of analyses, blood, nucleic acids, cells, tissues, secretions/excreta, fixed histological material, organs and their fragments, bone marrow, isolated biologically active substances (e.g., proteins), pathogens/microorganisms/invertebrates and others. We assume that each unit could collect one or more types and subtypes of biological material. These results are presented in Fig. 2.

More than half, i.e., 35, out of the 60 units that completed the questionnaire collect blood samples. In this group, 6 biobanks reported collecting umbilical blood, 6 biobanks reported collecting blood products, 25 biobanks reported collecting blood components, and 33 biobanks did not specify the type of blood sample collected.

Cells were collected by 31 biobanks. These units collect somatic cells (17 biobanks), cell lines (16 biobanks), stem cells (14 biobanks), primary cells (9 biobanks), oncological cells (9 biobanks), modified cells (2 biobanks) or embryonic and other cell types (1 biobank).

Nucleic acids were collected by 30 of the identified biobanks; 26 of them collected DNA, and 21 collected RNA.

Solid tissue was collected by 27 identified biobanks. In this group, biobanks collect epithelial tissue (12 biobanks), connective tissue (8 biobanks), fat tissue (8 biobanks), muscle tissue (9 biobanks), processed fragments of human tissues (5 biobanks), nervous tissue (5 biobanks), bone tissue (3 biobanks), and cartilage tissue (3 biobanks), and 4 biobanks collect other types of tissues.

Different types of body fluids were collected by 19 biobanks. The most common sources are urine (10 biobanks), saliva (6 biobanks), milk (4 biobanks) and cerebrospinal fluid (4 biobanks). Other fluids of body cavities (2 biobanks), synovial fluid (1 biobank), and peritoneal fluid (1 biobank) were also stored in Polish biobanks. Five biobanks reported collecting other types of body fluids (not mentioned in our survey).

Among the units possessing human biological material, 15 biobanks collect fixed histological material, 11 units collect organs and their fragments, and 8 units collect bone marrow samples.

Protein isolates are collected by 7 biobanks. Out of these 7 units, 6 biobanks reported collecting total protein/mixtures of proteins, 2 antibodies, and 1 cytokines.

Four of the identified entities reported collecting pathogens/microorganisms obtained from the human body. All these units had bacterial collections; additionally, 2 of them had a collection of viruses, and 1 unit had a collection of fungi.

Five of the identified biobanks declared the collection of human biological material in a form that was not listed in the survey.

Furthermore, most identified biobanks that collect human biological material are focused on data linked to samples: 39 out of 60 reported collecting clinical and epidemiological data about sample donors (input information), and 34 biobanks indicated the storage of research data performed on their biological samples (data analysis results).

The other aspect that is important to characterize the Polish biobanking landscape is the collection size. Four respondents (7%) reported collections with more than 100,000 samples. There were 10 biobanks (17%) with collections of sample sizes ranging from 10,001 to 100,000 samples, 17 biobanks (28%) reported collections with 1001 to 10,000 samples, and 7 biobanks (12%) reported having 500 to 1000 samples. Most Polish biobanks (20 units, 33%) have collections of fewer than 500 samples. Two of the identified biobanks (3%) did not declare the size of their collections.

**Fig. 3:**
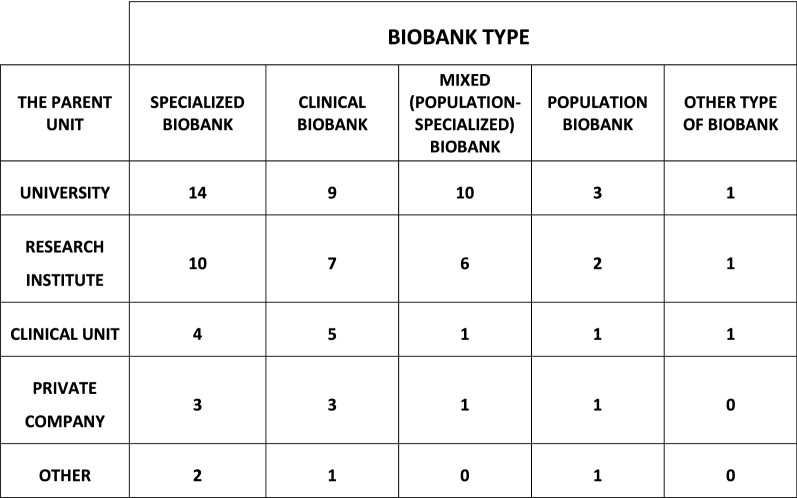
The number of Polish biobanks, their type, and the connection to the parent unit in the management structure. A single biobank could define itself as belonging to more than one type of biobank

The management structure and liaison of Polish biobanks are consistent with the European biobanking landscape. According to the obtained data, most biobanks are affiliated with universities (25 units, 42%), research institutes (18 units, 30%), clinical facilities (7 units, 12%), private companies (6 units, 10%) or other types of parent institutions (4 units, 7%). In addition, our aim was to characterize the types of biobanks in Poland. As stated before, we provided the possibility of using multiple-choice answers. Respondents could choose between population biobanks, specialized biobanks, mixed (population-specialized) biobanks, clinical biobanks or other types of biobanks. Analysis of the results showed that Polish biobanks are often undertaking collections in several fields. Figure 3 presents both the affiliation and the characteristics of the infrastructure declared in the questionnaire.

**Fig. 4:**
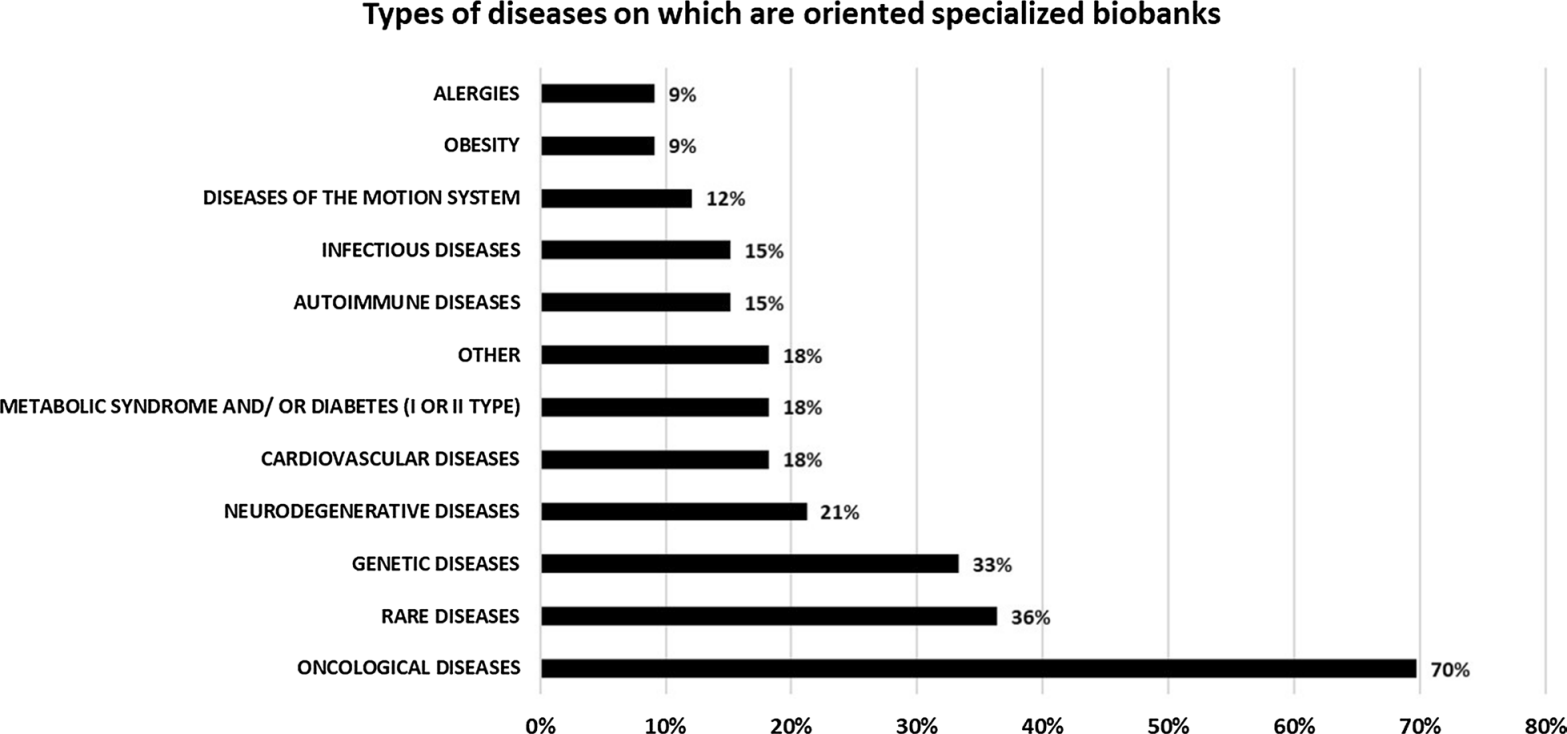
Types of diseases towards which specialized biobanks are oriented, gathering different types of biological material obtained from humans. The percentage was calculated in relation to the number of specialized biobanks analyzed (n = 33)

The collected data indicated that a considerable number of Polish biobanks are specialized (33 units), in contrast to population-based biobanks (8 units). These specialized types of biobanks are mostly focused on certain diseases. The largest portion of Polish specialized biobanks are interested in collections of samples from oncological diseases (23 biobanks), rare diseases (12 biobanks) and/or genetic disorders (11 biobanks), neurodegenerative diseases (7 biobanks), metabolic syndrome and/or diabetes mellitus type I or II (6 biobanks), cardiovascular diseases (6 biobanks), infectious diseases (5 biobanks), autoimmune diseases (5 biobanks), diseases of the motion system (4 biobanks), allergies (3 biobanks) and/or obesity (3 biobanks). Another 6 biobanks declared that they specialize in different/unspecified kinds of diseases (Fig. 4).

In addition, 25 units defined themselves as clinical biobanks concentrating mainly on transplants, blood transfusions or sample storage after the genetic diagnostic process. An additional 18 Polish biobanks described themselves as mixed, which means that they are a combination of population and specialized biobanks (Fig. 3). The results for mixed-type biobanks were similar to those for specialized biobanks—11 of them were concentrated mostly on oncological diseases (which represented 61% of all entities that defined themselves as mixed-type biobanks). Eight mixed-type biobanks declared a focus on cardiovascular diseases, obesity and autoimmune diseases (representing 44% of all entities that defined themselves as mixed-type biobanks). Seven mixed-type biobanks are focused on metabolic syndrome and/or diabetes mellitus type I or II and rare diseases (i.e., 39% of mixed-type biobanks). Fewer mixed-type biobanks are interested in genetic diseases (6 biobanks, 33%), neurodegenerative diseases (4 biobanks, 22%), infectious diseases (3 biobanks, 17%), diseases of the motion system (3 biobanks, 17%), and allergies (3 biobanks, 17%). From this group, 5 biobanks (28%) are focused on other/undefined types of diseases.

Surprisingly, only 8 units identified themselves as population biobanks that are monitoring public health regionally (there is no national initiative), and 3 biobanks declared their type as “other” (which means that the criteria mentioned in the survey did not cover their area of interest).

The *Information Survey* also included questions about the implementation of a quality management system (QMS). Most biobanks declared that their quality management system was in the process of being implemented (27 biobanks, i.e., 45% of all 60 units analyzed), 14 biobanks declared that the system had already been implemented (23%), and 11 units (18%) declared that they had nothing in place. Six units (10%) did not know the answer to this question, while the other 2 (3%) did not provide a response. This is consistent with responses to the question about the use of standard operating procedures (SOPs)—specialized procedures and instructions related to the processes occurring in the biobank. Most of the identified biobanks, i.e., 25 (42% of all 60 analyzed units) declared that they possess SOPs, 18 units (30% of all 60 units analyzed) were in the process of establishing them, and 13 units (22% of all 60 units analyzed) had prepared such procedures for part of their collections. Only 3 units (5%) had not yet prepared such procedures. One unit did not answer that question. At the same time, 57 (95%) of the identified units declared their willingness to participate in biobanking trainings and workshops organized by the BBMRI.pl Consortium (2 units did not answer the question, and 1 answered ‘I don’t know’).

Finally, we confirmed that biobanks in Poland are also ready to share their samples with other units. The declaration of cooperation was confirmed by 36 (60%) of the analyzed biobanks; only 4 units (7%) did not want to do so, and 18 (30%) units had not yet made a decision regarding sharing their own collections. Two (3%) units did not answer that question.

## Discussion

In the existing literature, no extensive analysis of the biobanking landscape in Poland had been performed thus far. Hence, this is the first report showing the diversity of biobanking units and thus also the scientific activities in Poland. First, our data indicate that there is a general trend towards establishing biobanks in the academic environment (72% of them) in contrast to the private sector (10%) (see Fig. 3). The highest number of identified biobanks in Poland are located at universities and research institutions. Second, biobanks have been identified at almost all medical schools and are often leading diagnostic centers for both civilization and rare diseases, as well as centers for clinical trials. For patients who participate in a variety of studies, access to well-equipped medical centers gives a sense of greater security and a sense of care, which strengthens confidence in developing medical sciences [1, 16, 20]. Additionally, we suspect that the linkage between biobanks and recognized academic centers may affect the popularization of the biological sample donation process. Although the donation of their own biological material for scientific purposes is not yet widely known and popular among Polish society, it has been shown that Poles will most willingly transfer their biological material to scientists associated with academic centers because they put the greatest trust in them, which is consistent with European trends [36]. The location of biobanks in academic centers, which are usually also associated with clinical hospitals in Poland, may increase the amount of biological material acquired in the future. Geographical access to biobanks is becoming simpler since they are found in over 80% of Polish regions (shown in Fig. 1). In the European landscape, the number of Polish biobanks is at an average level relative to the size of the country. According to the BBMRI-ERIC catalog, large EU countries, such as Finland or Norway, have fewer biobanking units but very large collections, while other countries, such as Italy, France and Sweden, report a significantly larger number of biobanking units (68, 95, and 113, respectively) with very diverse collections [37].

The present survey demonstrates that many Polish biobanks are specialized units that are oriented towards specific disease types (see Fig. 3). The visible majority of identified specialized biobanks declared that they are mainly focused on malignancies and rare or genetic disorders (shown in Fig. 4). As we know, such a focus is often a consequence of financing research around declared diseases and clinical trials [6, 21, 34, 35, 38]. Biobanks that declared themselves as mixed type constitute another category that is oriented, similar to specialized biobanks, towards malignancies and rare disease, but they also collect samples related to cardiovascular disease, obesity, autoimmune diseases, and metabolic syndrome/diabetes. These units will probably play an important role in research on these diseases in the coming years due to the broad spectrum of samples that can be combined in different studies. It is very interesting that 18 biobanks (30%) in Poland collect samples connected with rare diseases. Making these collections public can increase the participation of Polish entities in projects related to these diseases while making them important partners in conducting research on rare diseases. Furthermore, research conducted in these biobanks can significantly affect the development of personalized medicine. A thorough analysis of biobanks collecting samples related to rare diseases, which will allow, among other things, in-depth knowledge about their collections, is currently under development.

Considering our data and the population of Poland (ca. 38 million citizens), there are not many population-based biobanks (8 units, 13%) dedicated exclusively to monitoring epidemiological health and the future occurrence of diseases affecting all of civilization or the development and transmission of severe viral infections, such as the recent spread of COVID-19 (see Fig. 3). However, it should be noted that population-type collections are also found in mixed biobanks (18 units, 30%), which in our survey were identified as units combining the features of specialized and population biobanks. Therefore, although the number of typical population biobanks in Poland is not very high, the number of population-type collections is already significant. In addition, the geographical distribution of these units throughout almost the entire Polish territory allows donors easy access to them and may in the future contribute to a significant increase in the population collection in Poland. We believe that our findings could be a great impetus for national funding bodies to strengthen the development of such units. BBMRI.pl will also actively promote across Europe those who collect samples, especially in the field of rare diseases, since a large number of such biobanks in Poland may accelerate the establishment of international collaboration and awards for scientific projects.

One of the most important aspects connected to the biobanking of biological material is the actual means of doing so. The survey showed that most biobanking units do not possess a specifically designated quality management system. However, the majority of them have developed and implemented their own procedures and instructions (SOPs) related to gathering, collecting, and processing biological material. A lack of QMS can mean that a major increase in the role of biobanks can be hindered because without a properly harmonized quality management system, joint research can be difficult or require the use of additional control points to confirm compliance with quality requirements [1, 14, 25]. It is important to design a properly working and harmonized quality management system, biobank interoperability, and substitutability to facilitate scientific discovery [39]. Our results indicate that Polish scientists do recognize that a QMS is essential in a biobank because 45% of them declare that they are in the process of implementing such a system. Additionally, 23% of Polish biobanks had already obtained it. It is worth noting that the presented data were collected mostly before the development of the quality management standards by the BBMRI.pl Consortium [8] and before or just after the ISO 20387:2018 *Biotechnology—Biobanking—General requirements for biobanking* norm [7] was published. The lack of specific guidelines on quality assurance methods for biobanking processes undoubtedly had an impact on limiting the number of biobanks with implemented QMSs in Poland. Despite these objective difficulties, however, almost 70% of Polish units try to ensure the quality of their samples. Considering quality management systems, ELSI issues cannot be omitted. Polish biobanks are aware of the needs related to maintaining proper ethical and legal standards and meeting European standards to ensure the safety and privacy of donors and their data [9, 10]. Implementation of the BBMRI.pl project and the related continuous development of biobanking quality and ELSI standards, as well as their promotion in the scientific community, will certainly increase the number of implemented quality systems and ELSI solutions in Polish biobanks and will gradually improve their excellence. Moreover, most Polish biobankers are interested in improving their own quality systems and declare a willingness to participate in trainings and workshops related to this aspect.

An unexpected result of our analysis is the willingness to share their collections and cooperation with other units according to a visible majority of Polish biobanks. Such a result was not obvious, especially in the case of new branches of science and research development, and the topic of biobanking is still not very common in Poland. We hope that such willingness creates links between healthcare, academia, and industry, thereby facilitating novel avenues of research [20, 40]. The distribution of biobanks throughout the Polish territory, their connection with scientific and clinical units, and their involvement in research on rare diseases may contribute to an increase in the number of multicenter studies. At the same time, collections of biological material in these biobanks are also valuable material for international research.

The development of personalized medicine and omics research demand the use of high-quality biological samples, which can occur due to their fractionation and storage in technologically advanced units. This is why biobanking of human biological samples evolved from single collections of samples to repositories that are well structured, managed and organized and run by professionally equipped units and trained personnel [41]. Individual countries and research funding agencies have granted significant funds to improve the functioning of biobanks in Europe and Poland. One such funded project, BBMRI.pl, enabled us to develop the *Information Survey* to explore and characterize the landscape of Polish biobanks, the diversity of biological material collections and the expertise of Polish biobanking institutions.

During the process of data collection, some limitations were encountered. The study relied on the results obtained from a survey completed by voluntary units involved in the biobanking of biological material. Some of the identified entities did not complete the questionnaires at all or filled them in only partially. Nevertheless, the collected results come from different types of biobanks located all over the country and allow us to obtain a valuable overview about the situation of Polish biobanks. All results presented in this work are a part of a large project and represent the situation of biobanks in Poland before the PBN was organized and at the beginning of active Polish cooperation with the BBMRI-ERIC. It will be very interesting to monitor how the situation changes within a couple of years.

## Conclusions

The data in this study indicate that a significant number of biobanking units have recently been established in Poland. Some biobanks have very diverse and unique collections of human biological materials, which may become an important element in future international projects. Most importantly, such biobanks can significantly affect the development of global knowledge in the field of selected rare diseases. Many Polish biobanks are specialized units that are oriented towards specific disease types. Although the number of population biobanks in Poland is not very high, the number of population-type collections is already significant. Although most biobanking units do not possess a specifically designated quality management system, our results indicate that Polish scientists do recognize that a QMS is essential in biobank operations; 45% of them declare that they are in the process of implementing a QMS. At the same time, scientists working in Polish biobanks are very open to scientific cooperation and sharing their collections with the international scientific community. The distribution of biobanks throughout the Polish territory, their connection with scientific and clinical units, and their involvement in research on rare diseases may contribute to an increase in the number of multicenter studies.

The creation of the PBN can provide support that will facilitate the development of biobanks by providing them with tools for running biobanks such as quality standards, IT solutions, and support in ethical and legal matters. All of this has a very large influence on the dynamic development of biobanking in Poland, which can increase the credibility and visibility of Polish collections and facilitate interesting and significant scientific research.

## Data Availability

The datasets generated and/or analyzed during the current study are available from the corresponding author upon reasonable request.

## Abbreviations

BBs: Biobanks
BBMRI.pl: Biobanking and BioMolecular resources Research Infrastructure, Poland
BBMRI-ERIC: Biobanking and BioMolecular resources Research Infrastructure-Consortium European Research Infrastructure
BM: Biological material
ELSI: Ethical, legal, and social implications system
IT: Informatics system
PBN: Polish Biobanking Network
QMS: Quality management system
